# Utilization of 3D evaluation for assessing selective caries removal practice in pre-clinical dental students: a pilot study

**DOI:** 10.1186/s12909-024-05296-3

**Published:** 2024-03-15

**Authors:** Worachate Romalee, Nattira Suksudaj, Patchayaporn Doungkom, Ding-Han Wang, Ming-Lun Hsu, Piyaphong Panpisut

**Affiliations:** 1 College of Dentistry, National Yang Ming Chiao Tung University, Linong St, Beitou District, Taipei City, 112 Taiwan; 2 Faculty of Dentistry, Thammasat University, 99 M. 18, T. Klong Nueng, A. Klong Luang, Pathum Thani, 12120 Thailand; 3 Mechanical Metrology Department, National Institute of Metrology Thailand, T. Klong 5, A. Klong Luang, Pathum Thani, 12120 Thailand; 4 Thammasat University Research Unit in Dental and Bone Substitute Biomaterials, 99 M. 18, T. Klong Nueng, A. Klong Luang, Pathum Thani, 12120 Thailand

**Keywords:** Dental caries, Dental education, Dental students, Operative dentistry, Selective, Caries removal, Assessment

## Abstract

**Background:**

Practicing and assessment of selective caries removal techniques in dental students remain challenges in many dental schools. The aim of this study was to utilize a 3D assessment technique, within a designated acceptable range of deviation, to evaluate the tendency of dental students in performing selective caries removal (SCR). The correlation between 3D assessment results and the conventional rubric rated by an instructor was also determined.

**Methods:**

Fifth-year dental students (*n* = 61) performed the SCR task on 3D-printed teeth containing simulated deep caries lesions in occlusal and proximal surfaces. One instructor assessed the results using a conventional analytic rubric. The excavated teeth were additionally evaluated using 3D analysis software with the designated acceptable range of deviations (± 0.5 mm) from the standard cavities. The average root mean square (RMS) value, representing the deviation between student-prepared cavities and the predefined standard cavities, was recorded. A tendency towards over-excavation was defined for RMS values > 0.5 mm, and towards under-excavation for RMS values < 0.5 mm.

**Results:**

The mean (min-max) of RMS was 0.27 (0.18–0.40) for occlusal and 0.29 (0.20–0.57)for proximal cavities. A tendency of dental students toward over-excavation was observed in both occlusal (74%) and proximal cavities (87%). There was a moderate negative correlation between the RMS values and the traditional rubric scores for both occlusal (R^2^ = 0.148, *P* = 0.002) and proximal cavities (R^2^ = 0.107, *P* = 0.010).

**Conclusions:**

The 3D evaluation technique effectively revealed specific tendencies in dental students’ caries removal skills. The integration of computerized assessments with traditional methods could potentially assist the instructors in delivering more objective and specific feedback to students. Further research is encouraged to investigate the impact of this assessment technique on improving student performance in selective caries removal skills.

**Supplementary Information:**

The online version contains supplementary material available at 10.1186/s12909-024-05296-3.

## Background

Dental caries remains one of the most prevalent preventable chronic diseases [1]. The selective caries removal (SCR) technique has offered more favorable cost-effectiveness than the traditional total caries removal technique for deep caries lesions [2]. Hence, practicing the selective caries removal technique for pre-doctoral dental students is crucial before seeing real patients [3]. Various studies introduce 3D-printed teeth containing customized simulated caries lesions [4]. The traditional assessment method is commonly performed by an instructor, requiring protected time to provide specific feedback to individual learners. However, the subjectivity of different instructors may result in low consistency and standard of assessment [5, 6]. This may compromise students’ skill development and performance, which could increase stress and depression during dental program [7].

Several computerized dental assessment systems based on the 3D superimposition technique have been proposed as effective and reliable tools to minimize subjectivity among instructors [8, 9]. These systems were mainly adopted for various dental practice skills, such as dental anatomy wax-up [10], prosthodontics [11], and restorative dentistry [12]. For example, CEREC prepCheck (Sirona Dental Systems LLC, Charlotte, NC, USA), has been shown to offer higher consistency and accuracy compared to traditional evaluations by dental instructors [9]. However, the cost and the need for complex user training remain significant limitations for many dental schools [6]. Thus, a computerized evaluation system could serve as an adjunct tool for the instructor to provide more objective feedback and assessment to dental students [13]. Additionally, the software may not yet be specifically designed for assessing selective caries removal practice.

It was demonstrated that the 3D superimposition technique, which establishes a range of deviations using a 3D analysis software, enables the comparison of the accuracy of intraoral impressions of buccal shelf [14]. The acceptable range in this context is a defined threshold of dimensional accuracy or range of deviation [15]. Deviation patterns in the dimension beyond the threshold (trueness) may be considered unacceptable, whether in the positive or negative values [16]. It is expected that this approach could be applied to assess student’s performance in selective caries removal practice. This method could also potentially reduces the need for multiple specialized or task-specific 3D dental software, thereby lowering the financial burden for dental schools by allowing the use of a single 3D analysis software for multiple practices.

In our study, 3D files generated from an intraoral scanner were used to compare student-prepared cavities and the predefined standard cavities. The acceptable range of deviation was determined using 3D analysis software (Materialise 3-matic, version 14.0; Materialise, Leuven, Belgium). The objective was, therefore, to demonstrate the use of the 3D superimposition technique with a designated acceptable range of deviation for assessing the SCR training in the 3D-printed tooth containing simulated deep caries lesions. In addition, the correlation between the results from the 3D assessment and the conventional rubric grading conducted by a dental instructor.

## Methods

The 5th -year dental students at the Faculty of Dentistry, Thammasat University, Thailand, were selected and recruited for the current study. The ethical approval was obtained from The Ethical Review Subcommittee for Research Involving Human Research Subjects of Thammasat University, Thailand (ID: 039/2564; approval date:27 April 2021). The participants were informed about the purpose and the requirement of this study before voluntarily signing consent forms. In total, 61 dental students participated in this study.

A protocol for preparing the printed teeth containing simulated deep caries in occlusal and proximal cavities was provided in the previous study [4]. The students were instructed about the SCR technique with standard instruments and high-speed and low-speed burs before performing the task. Then, students received two printed first permanent mandibular molars containing simulated caries on occlusal and proximal deep caries lesions. The teeth were fit in a dental model (Nissin Dental Products Inc, Kyoto, Japan) in a phantom head. The time assigned for each task was 20 min. Then, the excavated cavity was assessed by a single instructor (Piyaphong Panpisut) using an analytical rubric. The criteria for assessing the outcome include cavity outline, depth, wall inclination, smoothness, and the amount of caries removal (Supplementary file).

To assess the quality of excavation using 3D analysis, all prepared cavities were scanned using an intraoral scanner (CEREC Primescan AC, DENSPLY Sirona, Charlotte, North Carolina, USA). They were then superimposed onto the standard cavities prepared by the evaluator using the 3D analysis software (Materialise 3-matic, version 14.0; Materialise, Leuven, Belgium). The standard cavities and the student-prepared cavities were aligned and superimposed using the semi-auto registration method, with the crown part serving as a superimposed reference. A part comparison analysis was conducted within the range from − 2 mm to + 2 mm. The acceptance of cavity preparation or the acceptable range of deviation must fall within a maximum tolerated deviation of ± 0.5 mm. This value was selected based on the ability to visually estimate the dimensions of a cavity preparation using a standard periodontal probe with a 1 mm scale [17, 18], which was commonly used by instructors in laboratory practice.

The deviation between the students’ prepared cavities and the standard cavities prepared by an instructor was reported as root mean square values (RMS). The deviation areas were visualized on the color maps presented on the standard models. The students who exhibited positive and negative values in total deviation were interpreted as having a tendency for over-excavation and under-excavation, respectively. The type of total deviation observed among students was categorized into two groups: negative total deviation and positive total deviation. The negative total deviation was recorded when the amount of over-excavation was greater than that of under-excavation, and vice versa for the positive total deviation.

The data were analyzed using a statistical software program (SPSS Statistics for Windows, v21.0; IBM, Armonk, NY, USA). Pearson’s correlation was used to determine a correlation between the traditional rubric grading method and the 3D evaluation. The significance value was set at *p* = 0.05. The variables used to analyze the correlation were the total score from every domain of the traditional method and the mean RMS from the 3D evaluation method.

## Results

The median RMS of the proximal cavity (0.29) was slightly greater than that of the occlusal cavity (0.27)(Table 1 However, no significant difference was detected between the RMS values of both the occlusal and proximal cavities (*P* = 0.0614). Students exhibiting a positive value in total deviation were interpreted as demonstrating a tendency towards over-excavation. Conversely, those with a negative value in total deviation were interpreted as having a tendency toward under-excavation (Table 2). The results suggested that there was a dominant tendency among students to over-excavate in both occlusal (73.8%) and proximal (86.9%) cavities.

**Table 1:** The result from using 3D analysis for comparing student’s prepared cavities (*n* = 61) with the standard cavity (RMS is root mean square)

Type of cavity	Deviation from the standard prepared cavities		
	Minimum deviation (mm)	Maximum deviation (mm)	RMS (Median, min-max)
Occlusal cavity	–1.92	1.97	0.27 (0.18, 0.40)
Proximal cavity	–1.34	2.49	0.29 (0.20, 0.57)

**Table 2:** The type of total deviation observed from students. The positive total deviation was recorded when the amount of over-excavation was greater than that of under-excavation, and vice versa for the negative total deviation

Cavities/type of deviation	Positive total deviation	Negative total deviation	Total
Occlusal cavities	45 (73.8%)	16 (26.2%)	61 (100%)
Proximal cavities	53 (86.9%)	8 (13.1%)	61 (100%)

**Fig. 1:**
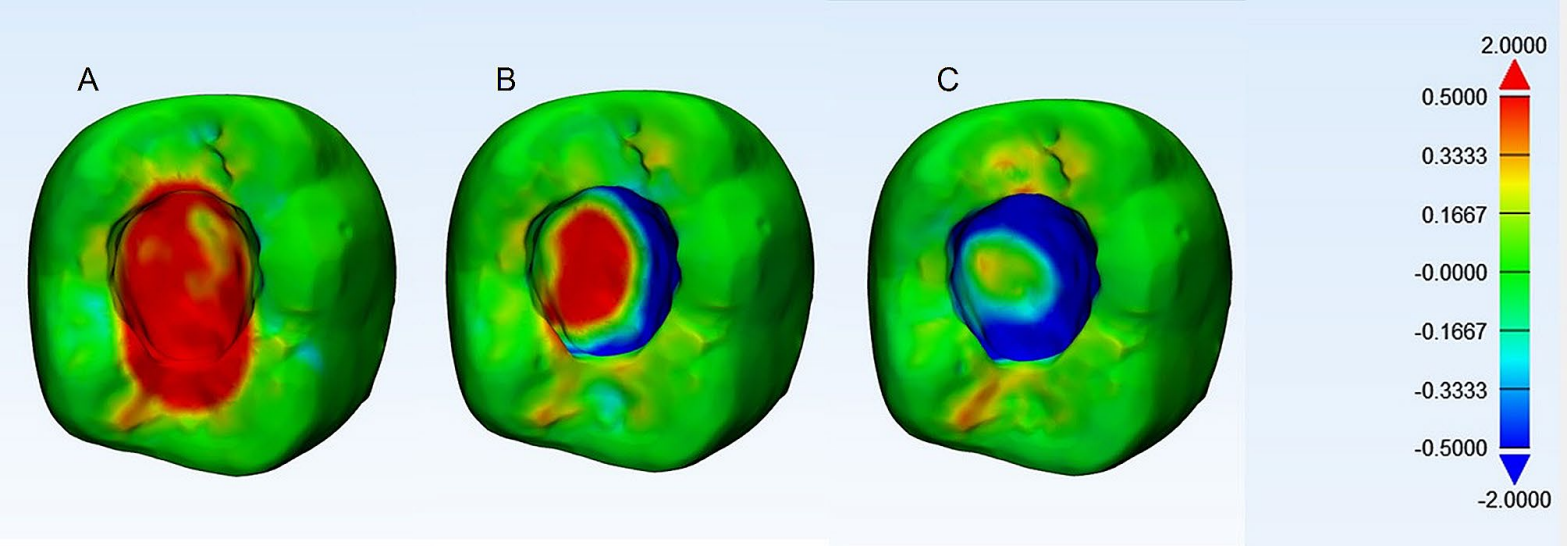
The 3D comparison results between the standard prepared occlusal cavities and the students’ prepared cavities. (**A**) A high tendency to over-excavation, (**B**) a cavity with minimal deviation, and (**C**) a cavity with a high tendency for under-excavation

**Fig. 2:**
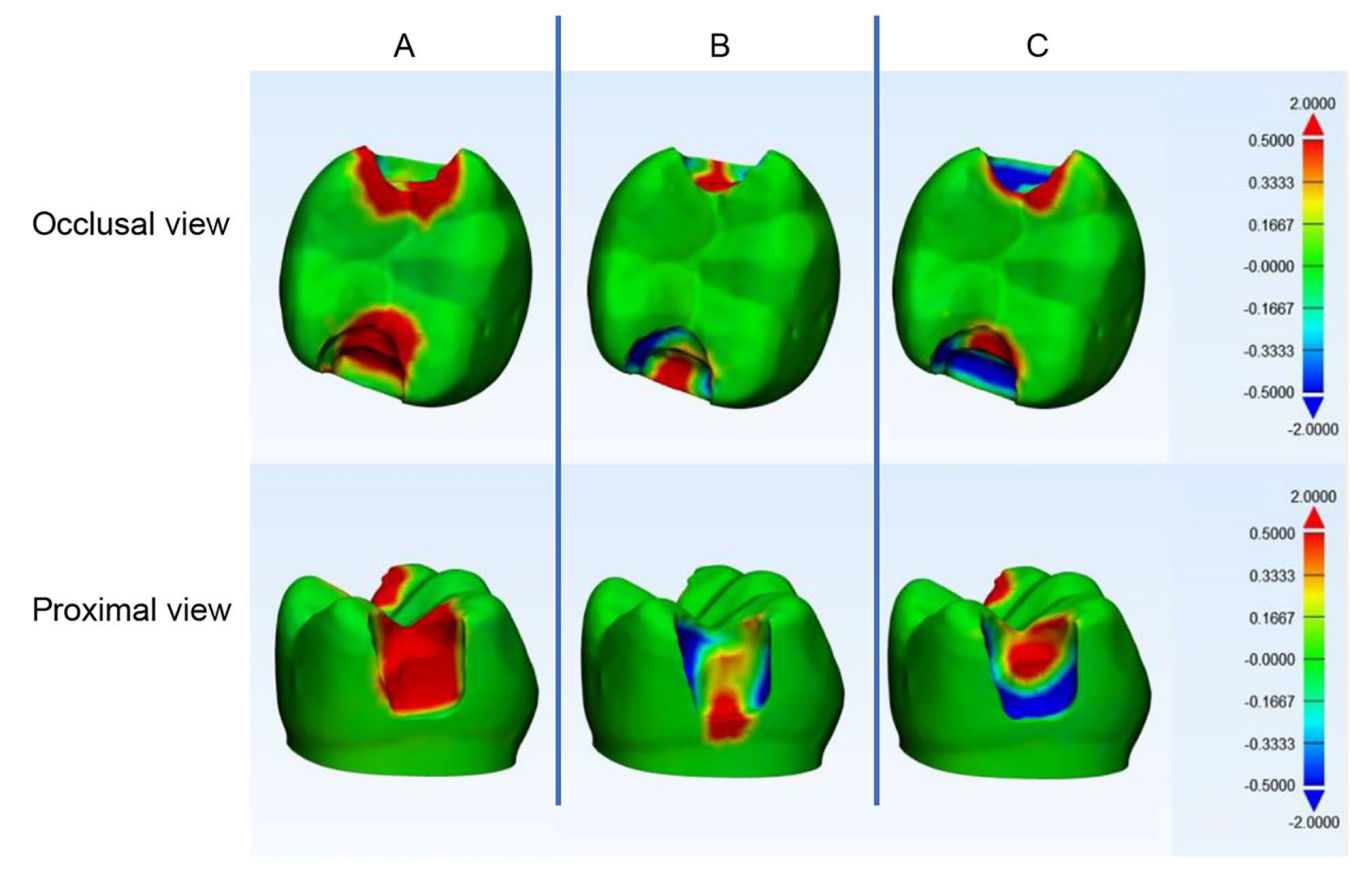
The 3D comparison between the standard prepared proximal cavity and the students’ prepared cavities. (**A**) a high tendency for over-excavation, (**B**) a low tendency for over-excavation or under-excavation, and (**C**) a high tendency for under-excavation

The example of superimposed data for occlusal and proximal cavities is presented in Figs. 1 and 2, respectively. The areas with no deviation were labelled as green. Positive deviations were displayed in red, representing that the excavation was larger than the tolerated deviation (+ 0.5 mm), whereas negative deviations were displayed in blue, representing under preparation from the tolerated deviation (–0.5 mm)(Figs. 1 and 2).

**Fig. 3:**
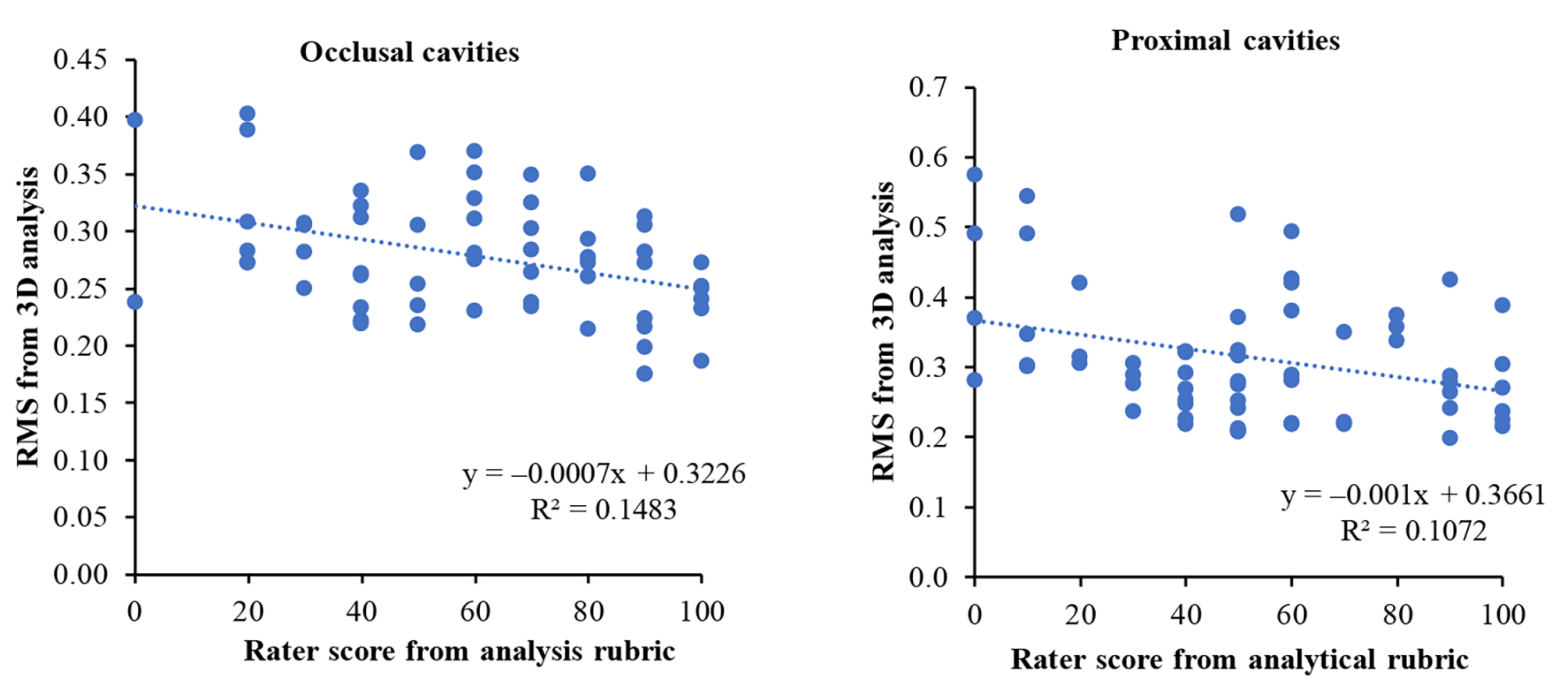
The negative correlation between RMS score versus rater score from an analytical rubric

Furthermore, a moderate negative correlation between RMS and the rating score obtained from the analytical rubric was observed in both occlusal and proximal cavities. Significant negative correlations were detected with Pearson’s correlation coefficients of − 0.385, R^2^ = 0.148, (*P* = 0.002) and − 0.327, R^2^ = 0.107, (*P* = 0.010) for occlusal cavities and proximal cavities, respectively (Fig. 3).

## Discussion

We expected that using printed teeth containing the customized deep caries lesions would enable students to practice similar tasks deliberately. The use of computerized assessment would provide tailored feedback for students. This could help improve students’ performance according to the deliberate practice model [19, 20]. This study aimed to examine the use of a 3D-computerized system with a designated acceptable range of deviation to assess a tendency of deviation in caries excavation for dental students.

The result of the current study indicated that the technique could detect the tendency toward over-excavation or under-excavation in dental students. Identifying the tendency of caries removal is vital feedback for dental students. For example, the feedback on the tendency toward under-excavation may increase the student’s awareness of the risk of insufficient removal of carious tissues in clinical practice. The remaining caries may cause trampoline effects [21, 22] that reduce the rigidity of the restoration. It is also known that affected dentine could not provide a reliable bond to adhesive materials [23]. On the other hand, the tendency toward over-excavation, especially in the pulpal area, would increase the risk of pulp exposure. This may increase the risk of pulpal infection and severe pain, requiring complicated treatments [24]. We expected this could be crucial specific information for instructors to provide feedback to students to enhance learning [25]. It is important to acknowledge that the instructor could also identify these tendencies. However, the present study had a limitation as it did not examine the correlation between the instructor’s results and the 3D analysis method. This issue should be addressed in future studies.

The SCR technique requires the tactile sensation of different dentin layers in caries lesions, which represents the degree of dentin demineralization and infection [26]. In the current study, we employed a low-stiffness resin-modified glass ionomer cement [27] mixed with the staining resin to mimic the caries lesions. These materials that have been polymerized may provide different tactile sensations when compared to the printed resin. However, they may not be able to offer the soft or leathery tactile sensation to replicate the actual caries lesion. This could compromise tactile feedback for students, thus resulting in the tendency toward over-excavation observed from students. Soft or gelatinous materials, such as injectable thermoplasticized gutta-percha or silicone, could be incorporated to mimic the soft or leathery texture of dentin in the upper layer of simulated caries lesions. This multilayer simulation may enhance students’ tactile feedback, aiding them in making more informed decisions about when to stop excavation to avoid pulp exposure. Additionally, improving the model’s fidelity could facilitate other endodontic practices, such as vital pulp therapy in cases of pulp exposure.

The current study demonstrated a moderate correlation between the score rated by an instructor and the degree of deviation by the digital assessment. This agreed with the previous study that compared the assessment between faculty members and digital assessment for tooth preparation [28]. The study reported a modest correlation between the two assessment methods, but the practical implications for clinical settings are not yet concluded. The negative correlation suggested that the low score from an instructor correlated with the RMS values It should be noted that the RMS value only represented the overall deviation and did not specifically indicate the specific criteria, as is the case in the analytical rubric. In order to balance the bias introduced by faculty assessments, computerized assessment was expected to be adopted in addition to traditional assessment. Future work should focus on correlating with multiple instructors to determine the systematic bias in faculty assessment. Moreover, the impact of 3D methods on the learning outcomes, particularly in terms of students’ competency in performing selective caries removal, should be investigated.

The current study set the acceptable deviation limit at 0.5 mm, based on the estimated visual accuracy of a standard periodontal probe (1 mm scale) used for self-assessment or giving feedback in a pre-clinical or early clinical year. It was expected that using the larger acceptable range of deviation initially would help increase self-confidence and motivation for dental students with limited experience. Then, the student’s SCR skill can be shaped and chained toward a more precise excavation and completion of all complex tasks, respectively [29]. Furthermore, we anticipated that the use of open-source 3D software for detailed analysis would facilitate dental instructors for other assessments/training, such as access opening, customized simple/complex cavity preparation, rest seat preparation, and waxing up occlusal anatomy.

The limitation of the 3D-computerized system with a designated acceptable range of deviation required specific software and an intra-oral scanner. The lack of a suitable scanner-to-student ratio could be considered the major limitation to employing computerized assessment techniques in the curriculum. The application of the photogrammetry technique using a digital camera or smartphone was introduced as the alternative and low-cost method to obtain digital 3D files for dental applications [30, 31]. The photogrammetry technique involves analyzing multiple photographs of an object, taken from various angles and processed using specialized software to create a 3D model. The software analyzes common points in these images to reconstruct the object’s shape and texture in three dimensions.

It was demonstrated that the accuracy of smartphones for photogrammetry techniques could range from 0.5 to 1.4 mm [32], which was lower than the level of accuracy in the 3D analysis software such as Prepcheck ($\sim$ 0.05 mm). It is worth noting that there is no established value for the accuracy that would be considered clinically meaningful. As a result, the use of smartphone scanners could be a promising alternative in the future. However, this method has some limitations at present, such as the requirement for high-resolution images which can vary depending on the model of the smartphone. Additionally, obtaining sufficient high-quality images for the analysis of shape and dimension requires consistent lighting and precise positioning of both the camera and the object.

## Conclusions

The 3D evaluation technique effectively identified specific tendencies in dental students’ caries removal skills. This study revealed a predominant tendency among students to over-excavate, observed in 73.8% of occlusal cavities and 86.9% of proximal cavities. Integrating computerized assessments with traditional methods can serve as a valuable tool for instructors, enabling them to provide objective and specific feedback to students. The potential impact of this approach on the improvement of the student’s performance should be further explored in future studies.

### Electronic supplementary material

Below is the link to the electronic supplementary material.

Supplementary Material 1

## Data Availability

The datasets used and/or analyzed during the current study are available from the corresponding author upon reasonable request.

## Abbreviations

SCR: Selective caries removal
RMS: Root mean square
