# Supplementary material for: Utilization of 3D evaluation for assessing selective caries removal practice in pre-clinical dental students: a pilot study
Source: BMC Med Educ. 2024 Mar 15;24:289. doi: 10.1186/s12909-024-05296-3 (PMC10941383; doi:10.1186/s12909-024-05296-3)
Supplement: Supplementary file 1 — Supplementary Material 1 [file 12909_2024_5296_MOESM1_ESM.docx]

**SupEvaluation form for cavity preparation**

| **Criteria/Level of performance** | **Unacceptable (0)** | **Good (10)** | **Excellent (20)** |
| --- | --- | --- | --- |
| **1. Outline of cavity** | Deviation from the carious outline > 2 mm | Slight deviation from the carious outline (within 0.5-2 mmm) | No deviation or deviate < 0.5 mm |
| **2. Depth of cavity** | - Exposure of pulp tissue or depth is too shallow  (pulpal depth < 2 mm) | Depth is slightly excessive, but no underlying red color of the pulp is visible | No visible coloration from the pulp |
| **3. Wall inclination** | Walls are either excessively divergent or convergent | Walls are slightly divergent or convergent | All walls are parallel |
| **4. Removal of caries** | Significant caries remains on the surrounding walls or all caries have been removed | Caries mainly present on the pulpal wall | Caries is strictly limited to the pulpal wall |
| **5. Smoothness of cavity** | Surface is rough with many sharp angles and edges | Mostly smooth with a few sharp angles | Walls are well-finished and smooth |
